# Invasive *Fusobacterium* nucleatum activates beta-catenin signaling in colorectal cancer via a TLR4/P-PAK1 cascade

**DOI:** 10.18632/oncotarget.15992

**Published:** 2017-03-07

**Authors:** Yongyu Chen, Yan Peng, Jiahui Yu, Ting Chen, Yaxin Wu, Lei Shi, Qing Li, Jiao Wu, Xiangsheng Fu

**Affiliations:** ^1^ Department of Gastroenterology, The Affiliated Hospital of Southwest Medical University, Sichuan, 646000, China

**Keywords:** Fusobacterium nucleatum, colorectal cancer, β-catenin signaling, toll-like receptor 4, p21-activated kinase 1

## Abstract

The underlying mechanism of *Fusobacterium nucleatum (Fn)* in the carcinogenesis of colorectal cancer (CRC) is poorly understood. Here, we examined *Fn* abundance in CRC tissues, as well as β-catenin, TLR4 and PAK1 protein abundance in *Fn* positive and *Fn* negative CRCs. Furthermore, we isolated a strain of *Fn (F01)* from a CRC tissue and examined whether *Fn (F01)* infection of colon cancer cells activated β-catenin signaling via the TLR4/P-PAK1/P-β-catenin S675 cascade. Invasive *Fn* was abundant in 62.2% of CRC tissues. TLR4, PAK1 and nuclear β-catenin proteins were more abundant within *Fn*-positive over *Fn*-negative CRCs (*P* < 0.05). *Fn* and its lipopolysaccharide induced a significant increase in TLR4/P-PAK1/P-β-catenin S675/C-myc/CyclinD1 protein abundance, as well as in the nuclear translocation of β-catenin. Furthermore, inhibition of TLR4 or PAK1 prior to challenge with *Fn* significantly decreased protein abundance of P-β-catenin S675, C-myc and Cyclin D1, as well as nuclear β-catenin accumulation. Inhibition of TLR4 significantly decreased P-PAK1 protein abundance, and for the first time, we observed an interaction between TLR4 and P-PAK1 using immunoprecipitation. Our data suggest that invasive *Fn* activates β-catenin signaling via a TLR4/P-PAK1/P-β-catenin S675 cascade in CRC. Furthermore, TLR4 and PAK1 could be potential pharmaceutical targets for the treatment of *Fn*-related CRCs.

## INTRODUCTION

A growing body of evidence suggests a complex link between gut microbiota, immunity, and colorectal carcinogenesis [1]. Recent studies show that a high amount of tissue-associated *Fusobacterium nucleatum (Fn)* has been connected to an advanced disease stage and poor clinical outcome in colorectal cancer (CRC) [1, 2]. However, the underlying mechanisms associated with this pathogenesis remain undetermined.

Recent studies reveal that *Fn*, a Gram-negative anaerobe, is associated with host immunity in colon carcinogenesis [3, 4]. It has been shown that *Fn* can stimulate the host immune response via toll-like receptor 4 (TLR4), which is a Gram-negative lipopolysaccharide (LPS) receptor [5].

It has been reported that high protein abundance of TLR4 in mouse intestinal epithelium induces colitis-associated tumor development [6], whereas TLR4 knock-out mice are protected against this disease [7]. In humans, high protein abundance of TLR4 is associated with more advanced grades of colonic neoplasia and lower overall survival [8, 9]. However, little is known about the exact TLR4 signaling cascade that is activated by gut microbiota in the colon.

It is well known that Wnt signaling pathway activation and β-catenin nuclear accumulation can be observed in approximately 90% of CRCs [10]. Mutations of the adenomatous polyposis coli (*APC*) or *β-catenin* genes are the main cause of β-catenin signaling activation in the majority of CRCs [10]. It was recently suggested that, in addition to genetic mutations, other signaling pathways and microenvironmental factors (such as gut flora) may be required to stabilize nuclear β-catenin [11, 12].

In recent years, it has been reported that p21-activated kinase 1 (PAK1) is associated with colon cancer progression and metastasis [13, 14]. PAK1 directly phosphorylates β-catenin at the Ser675 site, leading to a more stable and transcriptionally active β-catenin [15]. Interestingly, a recent study suggests that TLR4 can cause intestinal neoplasia through activation of the β-catenin signaling pathway [16]. Based on these findings, we hypothesized that *Fn* activates β-catenin signaling through a TLR4/PAK1 cascade in CRCs. A better understanding of signaling pathways activated by *Fn* may offer new opportunities to target the microbiota for CRC prevention and therapy.

To test this hypothesis, we examined whether *Fn* infection is associated with β-catenin nuclear accumulation in human CRC tissues and cells, and whether β-catenin accumulation by *Fn* is induced via the TLR4/PAK1 cascade. To our knowledge, this is the first study to examine the TLR4/PAK1 cascade activated by *Fn* in human CRC.

## RESULTS

### Invasive *Fn* is abundant in CRC tissues using fluorescence in situ hybridization (FISH)

**Figure 1:**
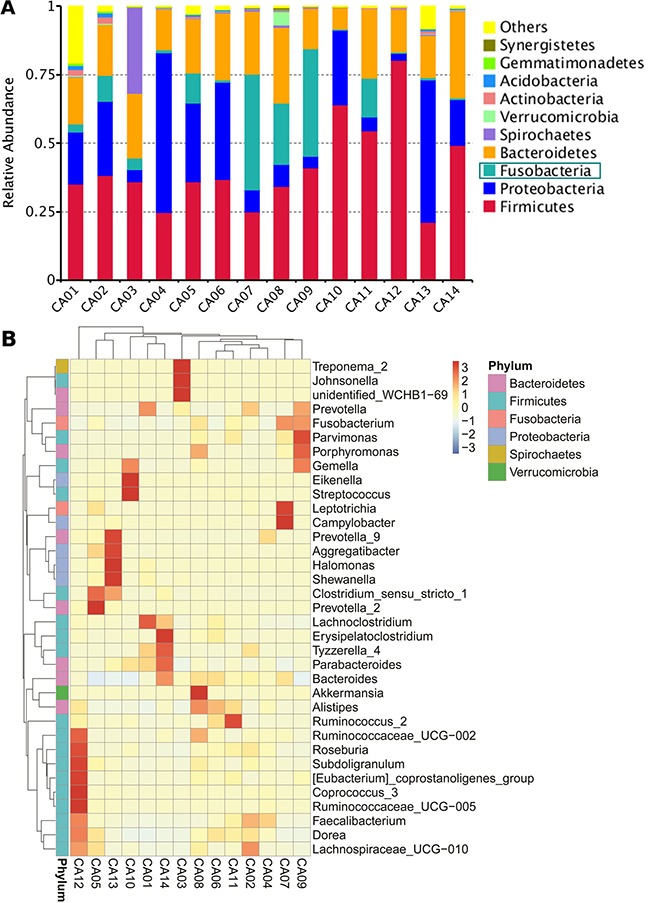
Biome analysis of human colorectal cancer samples **(A)** Relative abundance of phyla present in colorectal cancer samples (*n* = 14). Data shown represent the most abundant phyla, whereas low abundant and unclassified OTUs are grouped as “Others.” **(B)** Hierarchically clustered heat map analysis of the highly represented bacterial taxa (genus level) in 14 colorectal cancer samples. The relative percentages of the bacterial families are indicated by varying color intensities.

First, we investigated the bacterial community composition of 14 CRC tissues using 16S rRNA sequencing. *Firmicutes*, *Proteobacteria*, *Bacteroidetes* and *Fusobacteria* were the dominant phyla in most CRCs (Figure 1A). Hierarchically clustered heat map analysis of the highly represented bacterial taxa in the 14 CRCs is shown in Figure 1B. There was a great diversity in the bacterial composition among different CRC tissues. In a subset of CRCs, we detected a higher relative abundance of *Fusobacterium*, *Parvimonas*, *Porphyromonas*, and *Gemella*, which have been recently suggested as microbial pathogens potentially associated with CRC [17–19].

We then examined the abundance of invasive *Fn* in 98 CRCs using FISH. Positive detection of invasive *Fn* occurred in 61/98 (62.2%) of the CRC tissues. The clinicopathologic features of *Fn*-negative vs. *Fn*-positive lesions are shown in Table 1. There were no significant differences between *Fn*-negative and *Fn*-positive CRCs with respect to patient gender, age, histologic grade and lymph node metastasis (*P* > 0.05). However, *Fn* was detected at a significantly higher frequency in proximal CRCs than in distal CRCs (*P*= 0.045).

**Table 1:** Clinicopathologic characteristics in Fn-negative vs. Fn positive colorectal cancers

Fn abundance	Negative(n=37)	Positive(n=61)	P Value
**Gender**			
Female	16	24	0.703
Male	21	37	
**Age**			
(year)	59.4	58.0	0.281
(range)	36-81	40-74	
**Location**			
Proximal	16	39	0.045
Distal	21	22	
**Histologic grade**			
Well differentiated	10	12	0.519
Moderatlely differentiated	21	34	
Poorly differentiated	6	15	
**lymph node metastasis**	3	7	0.738

Fn, Fusobacterium nucleatum.

### Invasive *Fn* is associated with activated β-catenin signaling in CRC tissues

**Figure 2:**
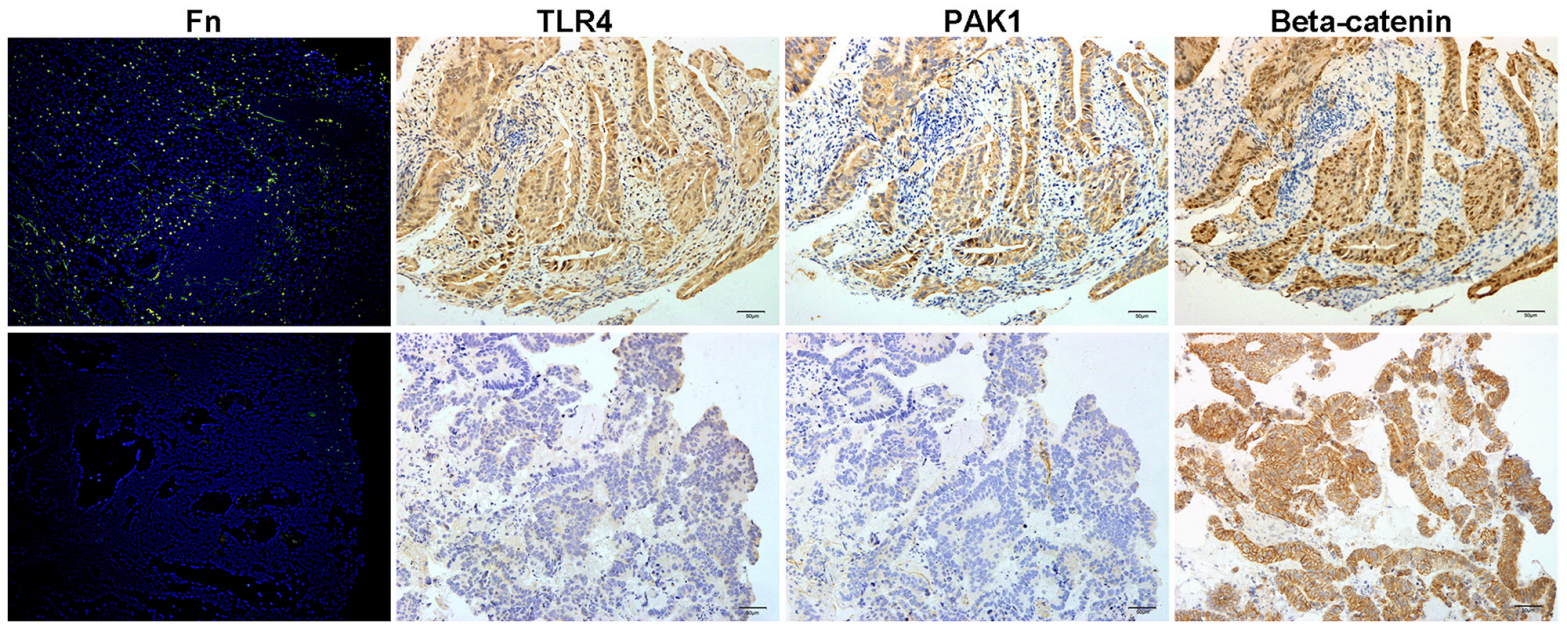
Invasive *Fn* in CRC tissues is associated with an activated β-catenin signaling pathway and TLR4/PAK1 protein abundance The upper panel shows that abundant invasive *Fn* in CRC tissue is associated with a high abundance of TLR4 and PAK1 and with β-catenin nuclear accumulation. The lower panel demonstrates that both TLR4 and PAK1 are absent in a *Fn*-negative CRC, and that cytoplasmic but not nuclear accumulation of β-catenin was seen in the tissue. Invasive *Fn* was detected by fluorescence in situ hybridization. 200× magnification.

We compared β-catenin, TLR4 and PAK1 protein abundance in 30 *Fn*-positive and 30 *Fn*-negative CRCs. The nuclear accumulation of β-catenin was more frequent in *Fn*-positive CRCs (*P*=0.028), suggesting that activated β-catenin signaling in these CRCs is associated with *Fn* infection. Similarly, TLR4 and PAK1 proteins were more abundant in *Fn*-positive CRCs when compared to *Fn*-negative CRCs (*P*< 0.05) (Table 2, Figure 2). These findings support the hypothesis that *Fn* contributes to the carcinogenesis of CRCs by activating β-catenin signaling through the TLR4/PAK1 cascade.

**Table 2:** Beta-catenin, TLR4 and PAK1 expressions in Fn-positive vs. Fn-negative colorectal cancers

	Fn-negative (n=30)	Fn-positive (n=30)	P-value
Beta-catenin			
Plasm accumulation	25 (83.3)	28 (93.3)	0.424
Nuclear accumulation	3 (10.0)	10 (33.3)	0.028
TLR4			
Positive	10 (33.3)	25 (83.3)	0.000
Strong positive	4 (13.3)	14 (46.7)	0.005
PAK1			
Positive	23 (76.7)	30 (100)	0.011
Strong positive	7 (23.3)	13 (43.3)	0.100

Fn, Fusobacterium nucleatum.

### *Fn* activates β-catenin signaling in colon cancer cells

**Figure 3:**
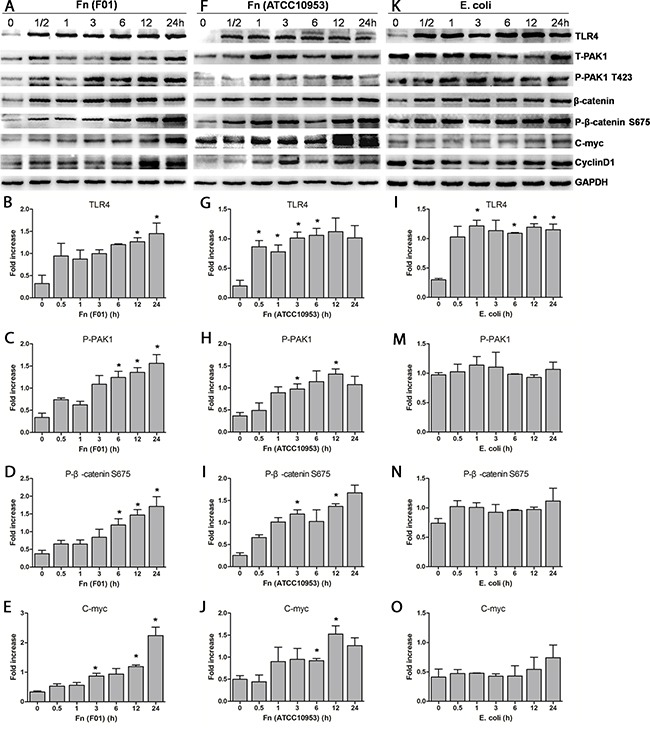
*Fn* activates the β-catenin signaling pathway in SW480 cells through the TLR4/P-PAK1/P-β-catenin S675 cascade **(A-E)** Western blots showing that the levels of TLR4, P-PAK1, P-β-catenin S675 and C-myc gradually increase when SW480 cells are challenged with *Fn (F01)* over increasing time periods. **(F-J)** The levels of TLR4, P-PAK1, P-β-catenin S675 and C-myc also gradually increase when SW480 cells are challenged with *Fn (ATCC10953)* over increasing time periods. **(K-O)** The levels of P-PAK1, P-β-catenin S675 and C-myc do not significantly increase when SW480 cells are challenged with *E. coli* for increasing time periods, although TLR4 protein significantly increases. T-PAK1, total PAK1; P-PAK1, phosphorylated PAK1. Bar diagrams represent the results obtained after densitometric scanning from three different experiments. Bars represent the mean ± SD. *, *P*< 0.05, compared with control group (0 h).

**Figure 4:**
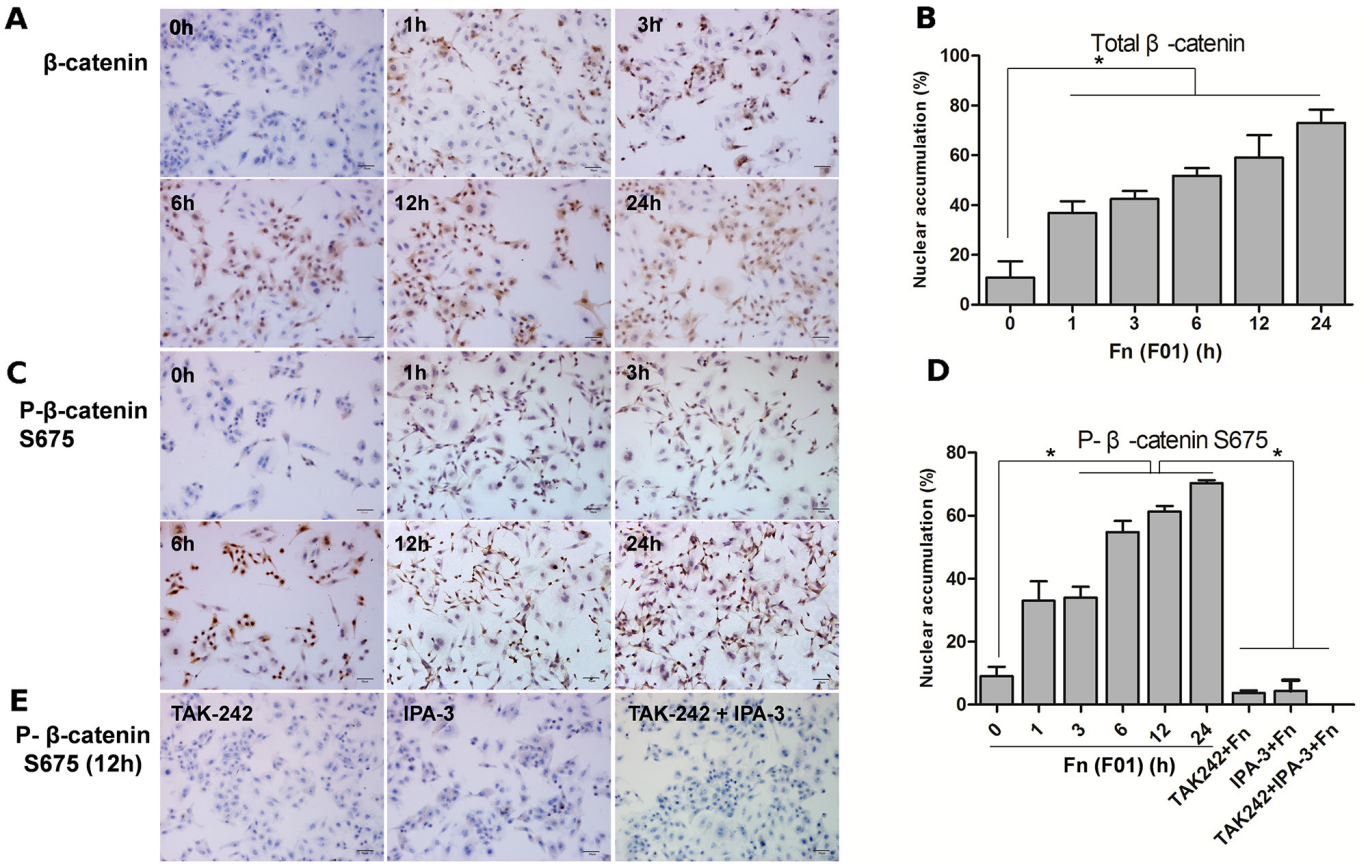
*Fn* activates nuclear accumulation of β-catenin and P-β-catenin S675 in SW480 cells **(A, B)** *Fn (F01)* significantly induces nuclear accumulation of total β-catenin over increasing time periods in SW480 cells. **(C, D)** *Fn (F01)* significantly induces nuclear accumulation of P-β-catenin S675 over increasing time periods in SW480 cells. **(D, E)** TAK-242 and IPA-3 inhibited the nuclear translocation of P-β-catenin S675 induced by *Fn (F01)* in SW480 cells. Three random 200× magnification fields per sample were evaluated, and the average percentage of β-catenin nuclear accumulation per field was calculated. Bars represent the mean ± SD. *, *P*< 0.05.

We directly cultured *Fn* anaerobically from frozen tumor sections of a right-sided colon cancer and obtained a single isolate *Fn* (*F01*) (Supplementary Figure 1). The *Fn* (*F01)* sequence has been submitted to GenBank (accession number: SUB1766768 Seq01 KX692281). SW480 and Caco-2 cells were challenged with *Fn (F01)* to investigate the direct influence of invasive *Fn* on colon cancer cells. *Fn* (*ATCC10953*) from the oral cavity and nonpathogenic *E. coli* were used as controls. High protein abundance of β-catenin (total β-catenin and P-β-catenin S675) and its target genes, C-myc and Cyclin D1, were detected in SW480 cells (Figure 3A–3E, Supplementary Figure 2A-2C) and Caco-2 cells (Supplementary Figure 3) infected with *Fn* (*F01*). *Fn* (*F01*) challenge in SW480 cells stimulated the nuclear translocation of both total β-catenin and β-catenin S675 (Figure 4). *Fn* (*ATCC10953*) also stimulated the protein abundance of β-catenin S675 and its target genes C-myc and Cyclin D1 (Figure 3F–3J, Supplementary Figure 2D-2F). However, *E coli* seldom caused an increase in β-catenin S675, C-myc and Cyclin D1 protein abundance; although it significantly increased TLR4 abundance (Figure 3K–3O, Supplementary Figure 2G-2I). These results suggest that *Fn* from both colon cancer tissue and oral cavity origin can activate the β-catenin signaling pathway in colon cancer cells.

### *Fn* activates β-catenin signaling via LPS and involves TLR4/PAK1 cascade

**Figure 5:**
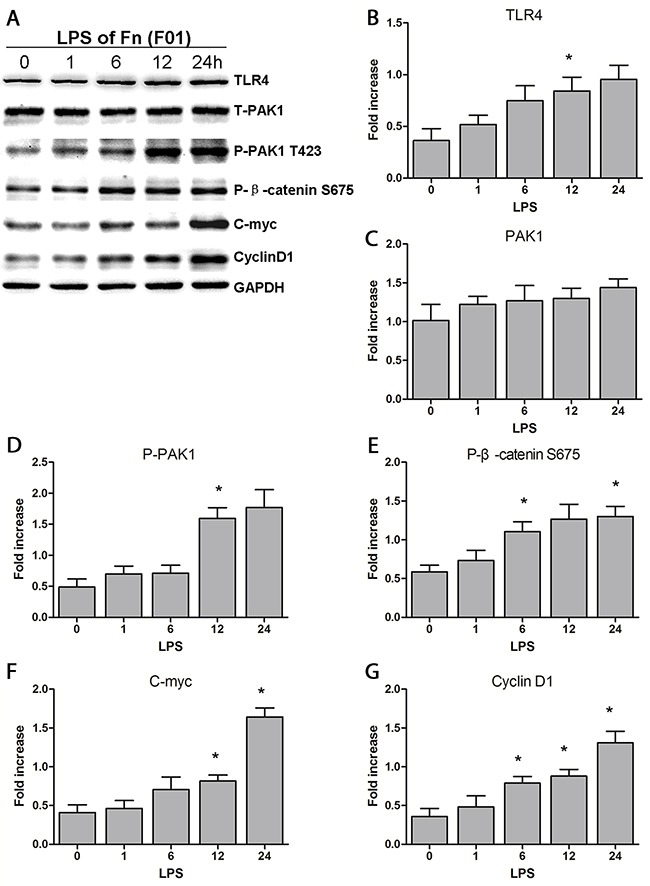
LPS of *Fn* could be the main reason for activation of the β-catenin signaling pathway through the TLR4/P-PAK1/P-β-catenin S675 cascade **(A-G)** Western blots showing that TLR4, P-PAK1, P-β-catenin S675, C-myc and Cyclin D1 levels gradually increase when SW480 cells are challenged with LPS extracted from *Fn (F01)* over increasing time periods. Bar diagrams represent the results obtained after densitometric scanning from three different experiments. Bars represent the mean ± SD. *, *P*< 0.05, as compared with control group (0 h).

To investigate if *Fn* activates β-catenin signaling through its LPS, we challenged SW480 cells with LPS extracted from cultured *Fn (F01)*. LPS induced high protein abundance of P-β-catenin S675 and its target genes C-myc and Cyclin D1, with similarly increasing trends (Figure 5). These findings indicate that the LPS of *Fn* is the main activator of β-catenin signaling in colon cancer cells.

Moreover, in cells challenged with *Fn (F01)*, *Fn* (*ATCC10953*) and the LPS of *Fn* (*F01*), elevations of both TLR4 and phosphor-PAK1 (P-PAK1) proteins levels began at 0.5h or 1h, and showed similar trends of growth over time (Figure 3, Figure 5). These findings indicate that activated β-catenin signaling by *Fn* infection in colon cancer involve a TLR4/P-PAK1 cascade.

### *Fn* activating β-catenin signaling might involve TLR4/P-PAK1 interaction

**Figure 6:**
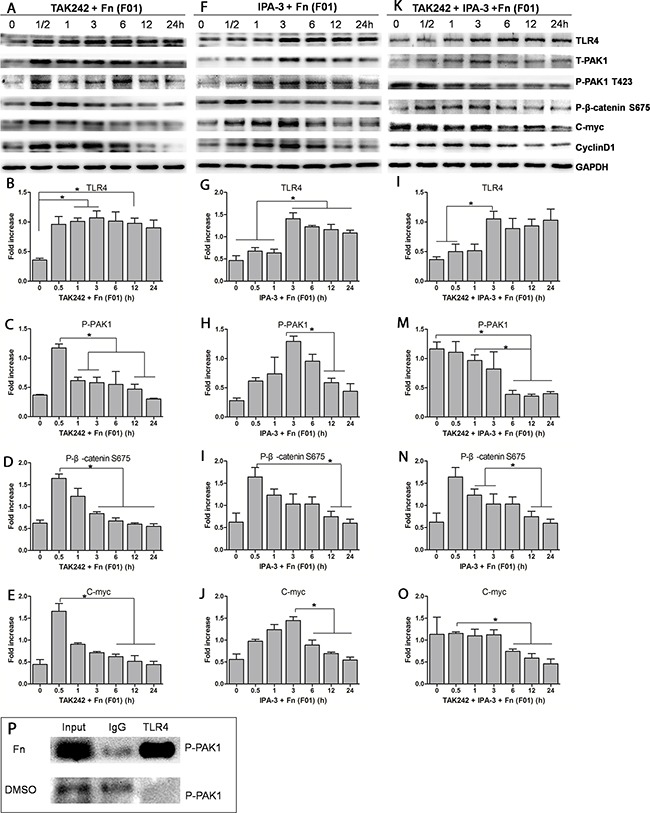
Activation of the β-catenin signaling pathway by *Fn (F01)* can be inhibited by both the TLR4 inhibitor (TAK-242) and PAK1 inhibitor (IPA-3) **(A-E)** Western blots showing that P-PAK1, P-β-catenin S675 and C-myc levels significantly decrease when SW480 cells are treated with TAK-242 prior to *Fn (F01)* challenge. **(F-J)** Western blots showing that P-PAK1, P-β-catenin S675 and C-myc levels significantly decrease when SW480 cells are treated with IPA-3 prior to *Fn (F01)* challenge. **(K-O)** Western blots showing that P-PAK1, P-β-catenin S675 and C-myc levels significantly decrease when SW480 cells are treated with both TAK-242 and IPA-3 prior to *Fn (F01)* challenge. **(P)** Cell lysates from *Fn (F01)* challenged SW480 cells were subjected to immunoprecipitation with anti-TLR4 agarose beads. DMSO was used as controls. Total lysates (Input), IgG and anti-TLR4 immunoprecipitations were subjected to western blots with P-PAK1 antibodies. Bar diagrams represent the results obtained after densitometric scanning from three different experiments. Bars represent the mean ± SD. *, *P*< 0.05.

An *Fn (F01*) induced TLR4 increase in SW480 cells did not change as a result of pretreatment with the TLR4 inhibitor TAK-242 or the PAK1 inhibitor IPA-3. However, pretreatment of SW480 cells with TAK-242 significantly decreased P-PAK1 protein abundance, suggesting that in this instance, P-PAK1 activation could depend on TLR4. Furthermore, pretreatment with either TAK-242 or IPA-3, or both, prior to challenge with *Fn (F01)* led to a significant decrease of P-β-catenin S675, C-myc and Cyclin D1 with similar trends (Figure 6A–6O, Supplementary Figure 4). Additionally, a significant decrease of nuclear β-catenin accumulation was observed (Figure 4D and 4E). These results suggest that the TLR4/PAK1 cascade has a role in *Fn*-challenged β-catenin signaling activation in colon cancer cells. Using immunoprecipitation (IP), we observed for the first time an interaction between TLR4 and P-PAK1 (Figure 6P), suggesting that TLR4 is possibly an upstream activator of P-PAK1.

## DISCUSSION

As a newly identified CRC-associated pathogen, the specific underlying pathogenic mechanism of *Fn*, is poorly understood. Here, we report that *Fn* activates the β-catenin signaling pathway via a TLR4/P-PAK1 cascade in the carcinogenesis of CRC.

It was recently argued that PAK1 activity is required for full β-catenin signaling in the carcinogenesis of CRC [15, 20]. PAKs are involved in the innate immune response, and accumulating evidence implicates PAK1 in colon inflammation and cancer [21]. Previous studies have shown that PAK1 can enhance β-catenin levels and activity through S675 phosphorylation in CRC cells [15]. Phosphorylation at Thr423 is critical for the maintenance of PAK1 activation [22]. Our study demonstrates that *Fn* significantly increases P-PAK1 protein levels, leading to β-catenin S675 phosphorylation and nuclear accumulation, whereas inhibition of PAK1 greatly decreases the level of P-β-catenin S675. This indicates that P-PAK1 is an upstream component necessary for maintaining *Fn*-challenged transcriptional activation of β-catenin.

We further investigated the possible mechanism by which *Fn* modulates PAK1/β-catenin signaling. A recent study reported that adhesin FadA, identified from *Fn*, binds to E-cadherin, thereby mediating *Fn* attachment of and invasion into colon cancer cells, subsequently resulting in activation of β-catenin signaling [4]. Another study shows that *Fn* invasion into host cells and cytokine production are independent of surface TLRs [23]. In the present study, however, *Fn* challenge significantly increased the abundance of TLR4 and P-PAK1 proteins. Pretreatment with TAK-242, a TLR4 inhibitor, prior to challenge with *Fn* led to the significant decrease of P-PAK1 abundance. Furthermore, a decrease in nuclear β-catenin accumulation, and reduced C-myc and CyclinD1 protein levels were also observed. These data suggest that P-PAK1/β-catenin signaling activation is likely dependent on TLR4. Furthermore, we observed for the first time an interaction between TLR4 and P-PAK1 using IP, suggesting that TLR4 is possibly the upstream element responsible for activating PAK1; however, the mechanisms of interaction between TLR4 and P-PAK1 require further investigation.

TLR4 recognizes the LPS of Gram-negative bacteria [24]. In the present study, LPS extracted from *Fn* (*F01*) significantly increased the protein abundance of TLR4, P-PAK1, β-catenin S675, C-myc and Cyclin D1. Moreover, β-catenin signaling can be inhibited by both TLR4 and PAK1 inhibitors. Collectively, our results indicate that *Fn* may primarily activate β-catenin signaling through its LPS, subsequently involving a TLR4/PAK1 cascade in colon cancer cells.

As a heterogeneous group of organisms, *Fn* is a common resident of both the oral cavity and the human gut mucosa [25]. In the present study, both a gut-derived *Fn* (*F01*) strain from a CRC patient and a *Fn (ATCC10953)* strain originating from the oral cavity was able to induce PAK1-mediated transcriptional activation of β-catenin. A recent study reported the abundance of some oral bacteria in the stool of patients with CRC, suggesting an oral-gut translocation route [26]. Thus, some oral bacteria such as *Fn*, can become opportunistic pathogens upon invasion of the gut microbiota, thereby indicating that improved oral hygiene may be useful in the prevention and treatment of CRCs. However, whether the present *F01* strain, originating from CRC tissue, displays a more invasive phenotype than those originating from the oral cavity or from healthy controls, requires further investigation. Moreover, our data show that TAK-242 and IPA-3 are very effective in blocking β-catenin signaling in colon cancer cells challenged by *Fn*. Therefore, TLR4 and PAK1 could be putative pharmaceutical targets for the treatment of *Fn*-related CRCs.

Recently it has been argued that instead of a single pathogenic bacterial species, it may be multiple species that contribute to CRC [27]. In our study, in addition to *Fn*, we found that *Porphyromonas*, *Parvimonas*, and *Gemella spp*. were also abundant in some CRC tissues. Other large-scale studies have identified an over-abundance of *Fn* within CRC tissues. Furthermore, they have observed a significant co-occurrence of *Fusobacterium, Leptotrichia* and *Campylobacter* species [28,29]. The presence of these pathogens in CRCs could be attributed to a multi-species environment. Moreover, these other microbial species may be contributing to the potentiation of tumorigenesis and the β-catenin signaling.

Taken together, our data suggest that invasive *Fn* activates β-catenin signaling via a TLR4/P-PAK1/P-β-catenin S675 cascade in the carcinogenesis of CRC; TLR4 and PAK1 could be potential pharmaceutical targets for the treatment of *Fn*-related CRCs.

## MATERIALS AND METHODS

### Sample collection

All tissue samples were obtained from patients undergoing colonoscopy or surgery at the Affiliated Hospital of Southwest Medical University (Sichuan, China) between February 2015 and December 2015. Fresh CRC tissue specimens (n= 14) were obtained for bacterial 16s rRNA amplification and analysis, with 7 of the samples collected from the proximal location and 7 from the distal location. The average age of the 14 CRC patients was 57.2 years. Formalin-fixed, paraffin-embedded CRC tissues (n= 98) were obtained from the pathology department archives of the same hospital for FISH analysis and immunohistochemical staining. Clinicopathologic data for each patient was obtained from the hospital records. Informed consent was obtained from all participants and the project was approved by the institutional review board.

### Cell culture and reagents

The human colon cancer cell lines SW480 and Caco-2 were grown in DMEM high glucose, supplemented with 10% fetal bovine serum (FBS, Bovogen), 100 U/mL penicillin, and 0.1 mg/mL streptomycin (Beyotime, china) in a 37°C, 5% CO_2_ controlled incubator. A stock solution of the TLR4 antagonist TAK-242 (MedChemexpress, USA) was prepared in DMSO, and then further diluted with DMEM to yield a final concentration of 1 μM. TLR4 was inhibited with 1 μM TAK-242 for 1 hour prior to *Fn* or LPS stimulation. PAK1 was inhibited by IPA-3 (MedChemexpress, USA) (10 μM final concentration in DMEM) for 1 hour prior to stimulation.

### DNA extraction, 16S rRNA amplification, sequencing and data analysis

Total genomic DNA was extracted from 4 mm^3^ fresh CRC samples using TIANamp Bacteria DNA Kit (Tiangen, China) according to the manufacturer's protocol. The DNA was diluted to a 1 ng/μL working stock using sterile water. 16S rRNA amplification, sequencing and data analysis were performed as previously described [30]. PCRs were conducted using the 515F/806R primer set [31], which amplifies the V4 region of the 16S rRNA gene.

PCR products were mixed in equidensity ratios. The pooled PCR products were purified with GeneJET Gel Extraction Kit (Thermo Scientific, USA). Sequencing libraries were generated using NEB Next® Ultra™ DNA Library Prep Kit for Illumina (NEB, USA) following manufacturer's recommendations, and index codes were added. The library quantity and quality was assessed using the Qubit@ 2.0 Fluorometer (Thermo Scientific, USA) and the Agilent Bioanalyzer 2100 system. Finally, the library was sequenced on an Illumina HiSeq platform, generating 250 bp paired-end reads. Sequences were analyzed using the QIIME [32] software package.

### Microbial FISH analysis

5-μm-thick sections were prepared using samples obtained from the pathology department archives and hybridized as described in our previous study [33]. The sequence of the FITC-labeled *Fn*-targeted probe, FUS664, was: 5′- CTT GTA GTT CCG C(C /T) TAC CTC -3′ [34]. Five random 200 × magnification fields per sample were evaluated by an observer blind to sample status; the number of bacteria per field was calculated. Cases were considered *Fn*-positive when FUS664 probe-associated bacteria were visualized, on average, more than 10 times per field.

### Immunohistochemical staining

Indirect immunohistochemistry was performed with formalin-fixed, paraffin-embedded tissue sections. Primary antibodies used in the study were as follows: TLR4 (1:100 dilution; Santa Cruz, CA), PAK1 (1:100; Cell Signaling), and β-Catenin (1:100; Cell Signaling). After application of the appropriate secondary antibodies, the labeled antigens were visualized by the development of brown pigment via a standard 3,3′- diaminobenzidine (DAB) protocol. Slides were then counterstained lightly with hematoxylin. Staining without primary antibody was performed as a negative control. Immunoreactivities were estimated as described in our previous report [35].

### Western blot and IP assay

Total cellular protein was isolated from cultured cells with a protein extraction solution (Beyotime, china). Proteins were subjected to 10% sodium dodecyl sulfate polyacrylamide gel electrophoresis (SDS-PAGE) and transferred to polyvinylidene fluoride (PVDF) membranes at 0.35 A (1 h, 4°C) using a wet-blotting apparatus (Bio-Rad). Membranes were blocked with 5% skim milk in PBST for 2 hours at room temperature, and then incubated overnight at 4°C with the primary antibodies diluted in PBST as follows: TLR4 (1:500), PAK1 (1:1000), phosphorylated-PAK1 Thr 423 (1:500; Santa Cruz), β-Catenin (1:1000), P-β-Catenin Ser 675 (1:1000; Cell Signaling), C-myc (1:1500; Proteintech), Cyclin D1 (1:200; Beyotime) and GAPDH (1:10000; Bioworld). Membranes were then incubated with appropriate secondary antibodies for 1 h at room temperature. Blots were quantified by densitometry using Quantity One 4.5.0 software (Bio-Rad Laboratories, Inc.).

The IP assay was conducted as previously described using an IP Kit (Beyotime, China) [36]. SW480 cells were stimulated with *Fn* (*F01*) and a total cell lysate was obtained. SW480 cells treated with DMSO were used as a control. Total protein was immunoprecipitated using an anti-TLR4 antibody (Santa Cruz) complexed to protein A/G. The immunoprecipitated proteins were then analyzed by western blot with an antibody to detect P-PAK1 T423.

### *Fn* culture and LPS extraction

*Fn* culture was performed as previously described [37]. Frozen 6 mm^3^ sections of proximal colon cancer tissue were thawed and immediately placed into 500 μL of sterilized PBS, washed to remove loosely associated bacteria, and then homogenized using a sterile mortar and pestle. This suspension was directly poured into fastidious anaerobe broth (FAB), supplemented with vancomycin (4 ug/mL) and neomycin sulfate (30 ug/mL), and then incubated for 1 d in a humidified anaerobic chamber (85% N_2_, 10% H_2_, and 5% CO_2_ at 37°C). One hundred milliliter aliquots of this broth were spread onto pre-reduced brain heart infusion agar (BHI) plates supplemented with 5% defibrinated sheep blood (DSB), josamycin (3 ug/mL), vancomycin (4 ug/mL), neomycin sulfate (30 ug/mL), norfloxaxin (1 ug/mL), haemin (5 ug/mL) and menadione (1 ug/mL). Plates were inspected every 2 days for growth, and all colonies were picked and streak-purified further onto pre-reduced BHI plates supplemented with haemin (5 ug/mL), menadione (1 ug/mL), and 5% DSB. Single colonies were examined by phase microscopy, looking for slender rods or needle-shaped cells that are characteristic of *Fn*. DNA was extracted from positively identified isolates using TIANamp Bacteria DNA Kit. Aliquots were used as template DNA for a PCR with primers and conditions as described by Park et al [38]. An amplicon size of 171 bp confirmed that the isolate belonged to *Fn*. Next, verified isolates underwent a PCR to partially amplify the 16S rRNA gene for sequencing and confirmation of speciation using the same DNA template; primers and conditions are as defined by Zhao et al [39]. This product (1499 bp) was sent to Sangon Biotech (Shanghai, China) for sequence analysis, and obtained traces that confirmed *Fn* as the species. The isolated strain was obtained from the tumor specimen from patient number 01, and thus named *F01*. The strain was stored at −80°C in FAB + glycerol (50% v/v).

LPS extraction from the *Fn* culture was performed using an LPS extraction kit (Intron Biotechnology) according to the manufacturer's instructions.

### Immunocytochemistry

The SW480 cells cultured on glass slides were treated with *Fn* (*F01*, MOI 1:100) for 12 hours. Cells were then fixed in 4 % paraformaldehyde for 20 min at 4°C. After being permeabilized by incubation with 0.5 % Triton X-100 for 15 min at room temperature, cells were incubated with 0.3% hydrogen peroxide in methanol for 10 min. The slides were incubated with primary antibodies β-Catenin (1:100) and P-β-Catenin S675 (1:100) for overnight at 4°C. Glass slides were incubated with secondary antibody for 1 h at 37°C. The labelled antigens were visualized by the development of brown pigment via DAB. Slides were then counterstained lightly with hematoxylin. Staining without primary antibody was used as a negative control.

Three random 200× magnification fields per sample were evaluated and the average percentage of β-Catenin and P-β-Catenin S675 nuclear accumulation per field were calculated respectively.

### Scanning electron microscopy (SEM)

Cultured *Fn* (*F01*) was fixed with 4% glutaraldehyde overnight at 4°C, dehydrated through a graduated series of ethanol, then spread on silver paper, critical point dried and coated with gold palladium. Specimens were prepared for viewing under a JEOL-6380LV emission scanning electron microscopy (JEOL, Japan).

### *Fn* (*ATCC10953*) and nonpathogenic *E. coli* culture

*Fn* (*ATCC10953*) was graciously supplied by Dr. Junqiang Jiang of the Affiliated Dental Hospital of Southwest Medical University, and nonpathogenic *E. coli* was isolated from healthy human stool for use as a control. *Fn* (*ATCC10953*) was incubated for 4 days in FAB under anaerobic conditions at 37°C. *E. coli* was cultured for 24 hours in Luria-Bertani broth at 37°C.

### Bacterial infection and LPS stimulation of colon cancer cells

*Fn* (*F01*) and *Fn* (*ATCC10953*) were cultured with FAB, supplemented with vancomycin (4 ug/mL) and neomycin sulfate (30 ug/mL) in the anaerobic system at 37°C for 4-5 days, until they reached an OD_540nm_ of 0.8, corresponding to 10^9^ CFU/mL. The bacteria were diluted to 10^7^CFU/mL, corresponding to a multiplicity of infection (MOI) of 100 [40].

The SW480 and Caco-2 cells were separately seeded in 6-well plates and incubated for 24 to 48 hours (until they reached 60% of the well) at 37°C. Cells were washed with PBS, and media was replaced with DMEM high glucose, supplemented with 10% FBS. The cells were then infected with bacteria (MOI 1:100) for 30 minutes, 1, 3, 6, 12 and 24 hours. For LPS stimulation, the SW480 cells were incubated with 0.1 ug/mL *Fn* LPS for 1, 6, 12 and 24 hours. No bacteria or LPS were added to the control plates. The plates were shaken gently and incubated at 37°C in an incubator with controlled 5% CO_2_. At each time point, media was removed and cells were washed twice with PBS. Total protein was extracted from cells for western blot.

### Statistical analysis

Data are presented as the mean and standard deviation for continuous variables and as proportions for categorical variables. Data were analyzed using one-way ANOVA, followed by Bonferroni test for multiple comparisons. Differences in categorical variables were determined by the Chi-square or Fisher's exact tests, as appropriate. Differences were considered significant if *P* < 0.05. All significance tests were two-tailed. All statistical tests were performed using SPSS software Version 13.0 (SPSS Inc., Chicago, IL, USA).

## SUPPLEMENTARY MATERIALS FIGURES


