# Supplementary material for: Invasive Fusobacterium nucleatum activates beta-catenin signaling in colorectal cancer via a TLR4/P-PAK1 cascade
Source: Oncotarget. 2017 Mar 7;8(19):31802–14. doi: 10.18632/oncotarget.15992 (PMC5458249; doi:10.18632/oncotarget.15992)
Supplement: Supplementary file 1 [file oncotarget-08-31802-s001.pdf]

Invasive *Fusobacterium nucleatum* activates beta-catenin signaling in colorectal cancer via a TLR4/P-PAK1 cascade

SUPPLEMENTARY FIGURES

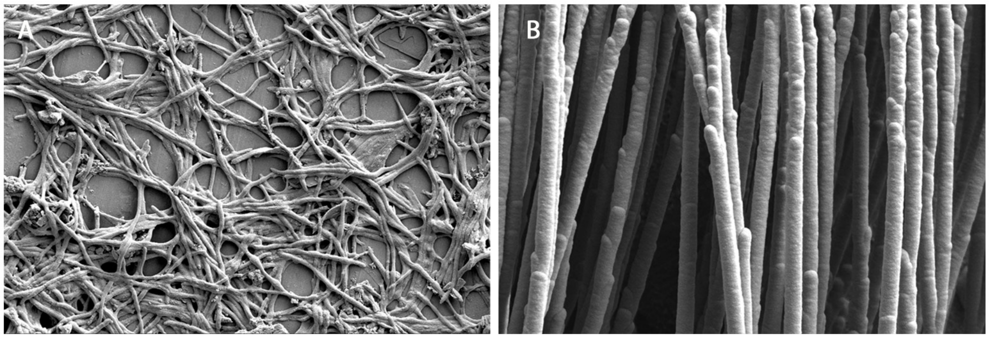

Supplementary Figure 1: Scanning electron microscopy shows the shape of cultured *Fn*. The strain was isolated from frozen tumor sections of a right-sided colon cancer and named *F01*. **(A)** 5,000× magnification; **(B)** 20,000× magnification.

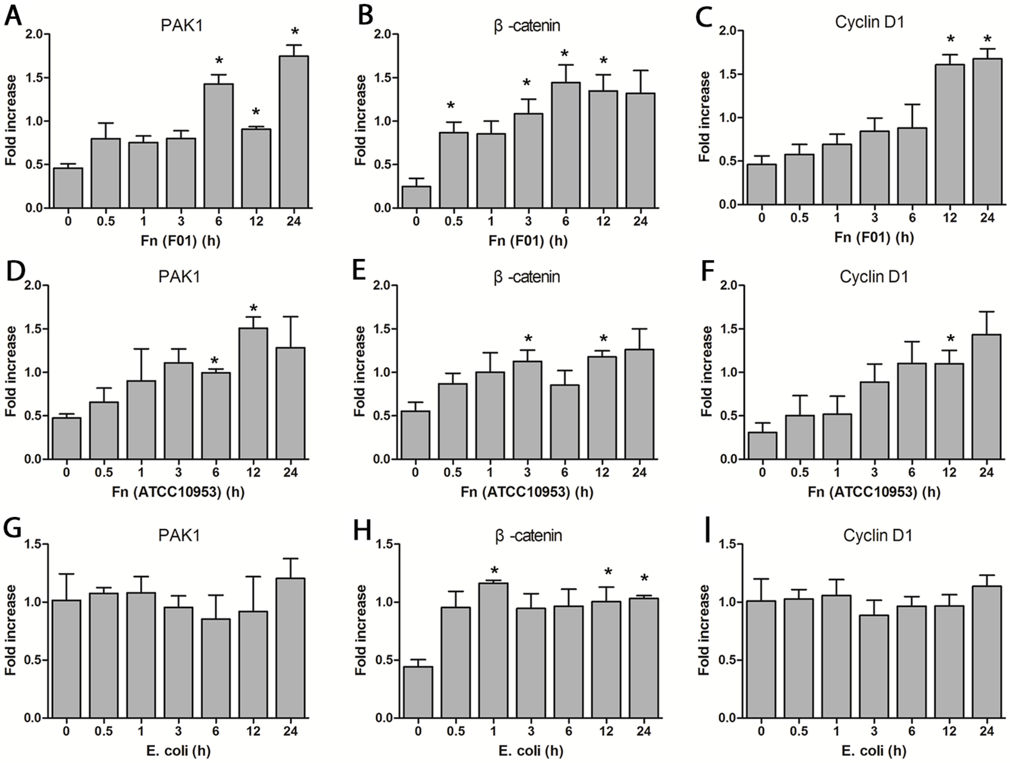

Supplementary Figure 2: *Fn* activates the β-catenin signaling pathway possibly through the TLR4/P-PAK1/P-β-catenin S675 cascade. **(A-C)** Western blots showing that the levels of PAK1, β-catenin and Cyclin D1 gradually increase when SW480 cells are challenged with *Fn (F01)* over time. **(D-F)** The PAK1, β-catenin and Cyclin D1 levels also gradually increase when SW480 cells are challenged with *Fn (ATCC10953)* over time. **(G-I)** The PAK1 and Cyclin D1 levels do not significantly increase when SW480 cells are challenged with *E. coli* for increasing time periods, although the protein abundance of total β-catenin significantly increases. Bar diagrams represent the results obtained after densitometric scanning from three different experiments. Bars represent the mean ± SD. *, $P< 0.05$, as compared with control group (0 h).

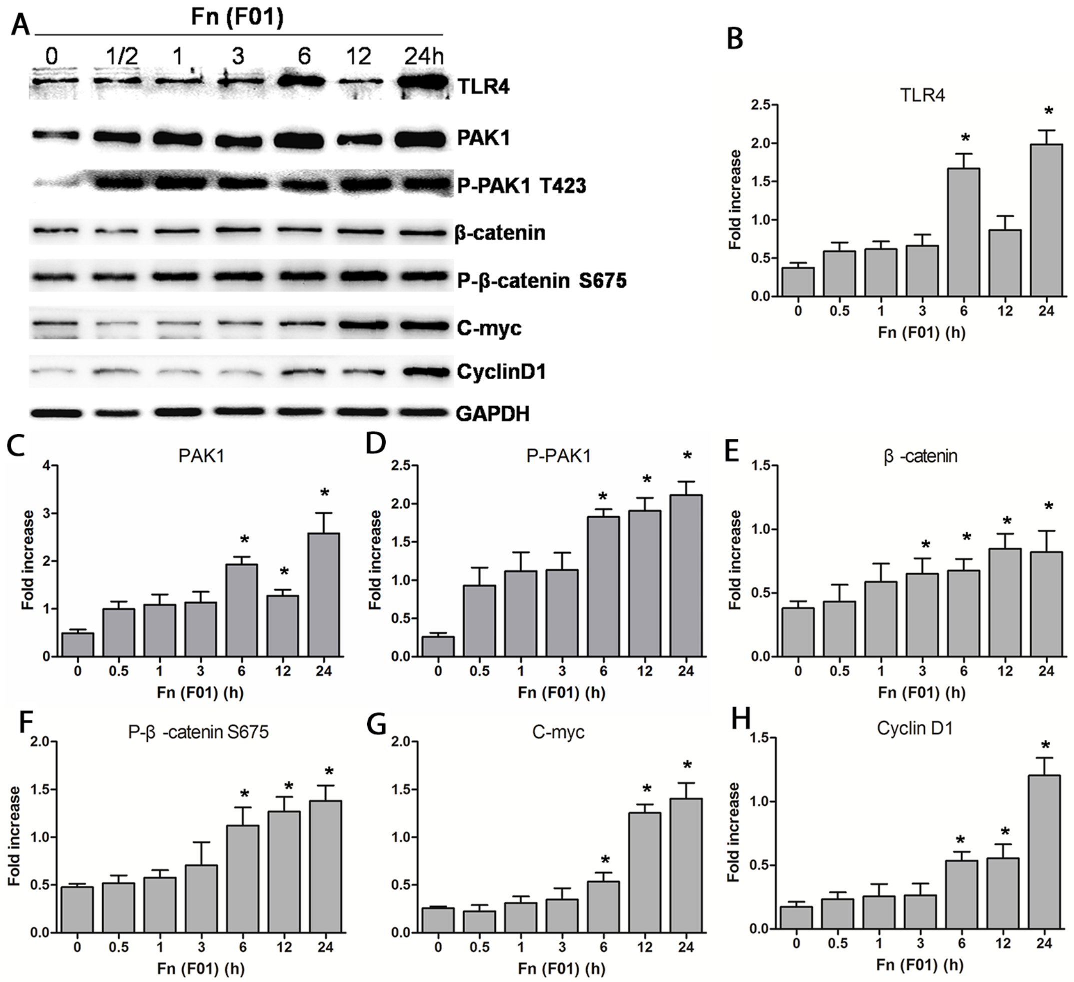

Supplementary Figure 3: *Fn (F01)* activates the β-catenin signaling pathway in Caco-2 cells through the TLR4/P-PAK1/P-β-catenin S675 cascade. (A) Western blots showing that the protein levels of TLR4, total PAK1, P-PAK1, total β-catenin, P-β-catenin S675, C-myc and Cyclin D1 increase with similar trends when Caco-2 cells are challenged with *Fn (F01)* over increasing time periods. **(B-H)** Bar diagrams represent the results obtained after densitometric scanning from three different experiments. Bars represent the mean ± SD. *, $P < 0.05$, as compared with control group (0 h).

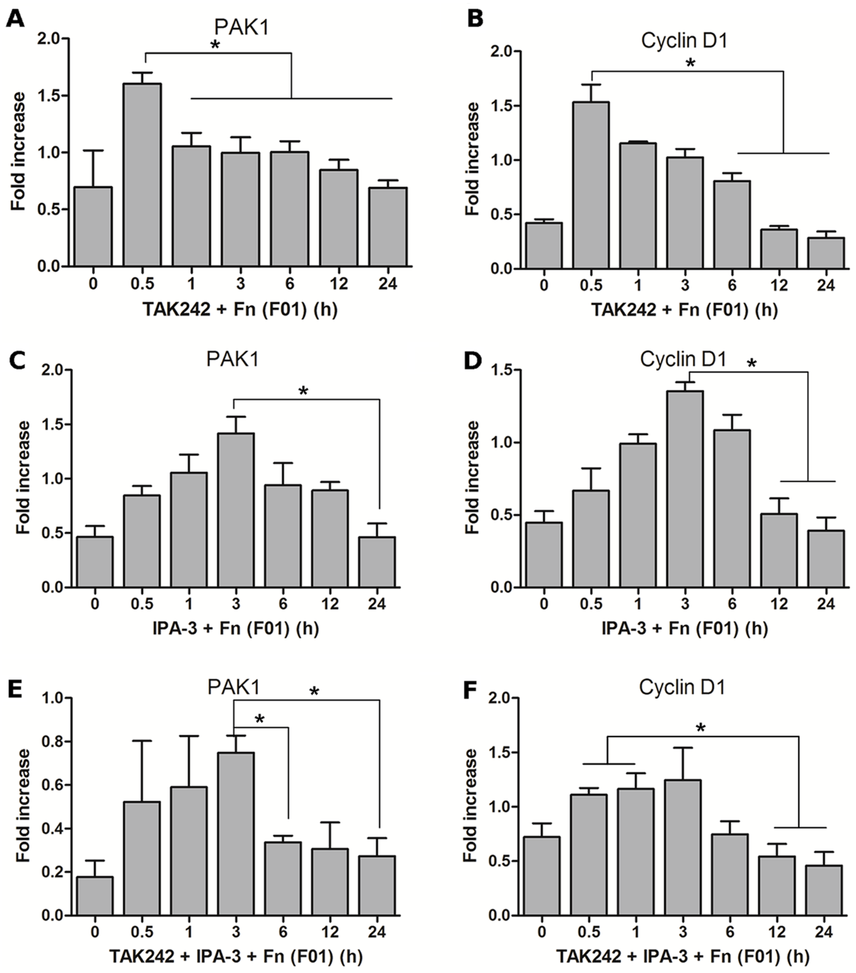

Supplementary Figure 4: Activation of the β-catenin signaling pathway by *Fn (F01)* can be inhibited by both the TLR4 inhibitor (TAK-242) and PAK1 inhibitor (IPA-3). **(A, B)** Western blots showing that PAK1 and Cyclin D1 levels significantly decrease when SW480 cells are treated with TAK-242 before *Fn (F01)* challenge. **(C, D)** Western blots showing that PAK1 and Cyclin D1 levels significantly decrease when SW480 cells are treated with IPA-3 before *Fn (F01)* challenge. **(E, F)** Western blots showing that PAK1 and Cyclin D1 levels significantly decrease when SW480 cells are treated with both TAK-242 and IPA-3 before *Fn (F01)* challenge. Bar diagrams represent the results obtained after densitometric scanning from three different experiments. Bars represent the mean ± SD. *, $P< 0.05$.
